# Human Umbilical Cord Blood-Derived Mesenchymal Stem Cell Transplantation for Patients with Decompensated Liver Cirrhosis

**DOI:** 10.1007/s11605-022-05528-1

**Published:** 2023-01-26

**Authors:** Zepeng Li, Xiaoling Zhou, Lu Han, Meijie Shi, Huanming Xiao, Ming Lin, Xiaoling Chi

**Affiliations:** 1 The Second Clinical College of Guangzhou University of Chinese Medicine, Guangzhou, 510006 Guangdong China; 2 Liuzhou Traditional Chinese Medical Hospital, Liuzhou, 545001 Guangxi China; 3 Department of Hepatology, The Second Affiliated Hospital of Guangzhou University of Chinese Medicine, Guangdong Provincial Hospital of Chinese Medicine, Guangzhou, 510120 Guangdong China

**Keywords:** Stem cell transplantation, Decompensated cirrhosis, Propensity score matching, Long-term survival, Hepatocellular carcinoma, Retrospective cohort

## Abstract

**Background or Purpose:**

Although human umbilical cord blood-derived mesenchymal stem cell transplantation (HUCB-MSCT) resulted in a good short-term therapeutic effect on patients with decompensated liver cirrhosis (DLC), the long-term survival remained unclear. This study aimed to evaluate the impact of HUCB-MSCT on long-term outcomes in patients with DLC.

**Methods:**

This retrospective cohort study included hospitalized patients with decompensated cirrhosis in Liuzhou Hospital of Traditional Chinese Medicine between November 2010 and February 2013. The primary outcome was overall survival (OS). The secondary outcomes were 3-year and 5-year survival rates and the occurrence rate of hepatocellular carcinoma (HCC).

**Results:**

A total of 201 subjects were enrolled, including 36 patients who underwent HUCB-MSCT (SCT group) and 165 patients who did not (non-SCT group). After PSM (1:2), there were 36 patients in the SCT group and 72 patients in non-SCT group. The 3-year and 5-year survival rates of the two groups were 83.3% vs. 61.8% and 63.9% vs. 43.6%, and median OS time was 92.50 and 50.80 months, respectively. HUCB-MSCT treatment was found to be an independent beneficial factor for patient OS (hazard ratio = 0.47; 95% CI: 0.29–0.76; *P* = 0.002). There was no significant difference in the occurrence rate of HCC between the two groups (*P* = 0.410).

**Discussion or Conclusions:**

HUCB-MSCT may improve long-term OS without increasing the occurrence of HCC in patients with DLC.

**Trial Registration:**

The Chinese Clinical Trial Registry (ChiCTR2100047550).

## Introduction

Cirrhosis is a diffuse liver disease, which is pathologically characterized by the occurrence of significant fibrosis and the formation of pseudolobules.^1^ Despite considerable research effort, there is a lack of acceptable treatment options for cirrhosis.^2^ Cirrhosis progresses slowly and is caused by several conditions including chronic alcoholism, hepatitis B virus and hepatitis C virus infection, and nonalcoholic steatohepatitis.^3^ When liver cirrhosis progresses to the decompensated stage, liver transplantation is the ultimate effective treatment. However, the clinical application of liver transplantation is severely limited due to the shortage of donor livers, high cost, and risks of surgical injury and immune rejection.^4^ New treatments are urgently needed as cirrhosis is a leading cause of morbidity and mortality worldwide.^5^

Basic and clinical studies in recent years have shown that stem cell therapy has the potential to become an effective alternative therapy for the treatment of liver cirrhosis.^6–8^ Mesenchymal stem cells (MSCs) are the most widely studied stem cells,^9,10^ both experimentally and clinically, and can be obtained from many tissues including the bone marrow, umbilical cord blood, adipose tissue, and placenta.^11–13^ The use of bone marrow derived stem cells is limited by the invasiveness of the procedure and the fact that the quality of derived stem cells was dependent upon age. Compared to bone marrow stem cells, the collection of human umbilical cord blood derived mesenchymal stem cells (HUCB-MSCs) is less invasive and carries less risk to the patient.^14^

Prior studies have shown that HUCB-MSCs can safely and effectively improve liver function as shown by improvements to Child-Turcotte-Pugh (CTP) and model for end-stage liver disease (MELD) scores in patients with liver cirrhosis.^15,16^ However, there is a lack of data on the long-term efficacy of umbilical cord mesenchymal stem cell transplantation, particularly in patients with liver cirrhosis. Moreover, transplantation of stem cells also carries safety risks such as the potential for tumor development.^17^ There are currently too few data including long-term follow-up to support the long-term safety of HUCB-MSC transplantation in liver cirrhosis patients.

This study aimed to evaluate the impact of HUCB-MSCT on long-term outcomes of patients with decompensated liver cirrhosis (DLC).

## Methods

### Study Design and Population

This retrospective cohort study included hospitalized patients with DLC admitted to Liuzhou Hospital of Traditional Chinese Medicine (Liuzhou, China) between November 2010 and February 2013. The study was approved by the Institutional Ethics Committee of Liuzhou Hospital of Traditional Chinese Medicine. The requirement for informed consent was waived by the committee due to the nature of the retrospective study.

The inclusion criteria were: (1) diagnosis of DLC; (2) aged between 18 and 75 years. Considering that some factors may affect the objective interpretation of the data analysis, the exclusion criteria were set as follows: (1) diagnosis with another serious disease outside the liver; (2) recipient of a late stage liver transplantation; (3) diagnosis of liver cancer before treatment; and (4) incomplete data or loss to follow-up. DLC was diagnosed by previous medical history, clinical manifestation, blood and imaging examinations, or liver biopsy.

### Data Collection

Clinical patient data, including age, sex, cause of disease, CTP score, CTP class, MELD score, and laboratory examination results (alanine aminotransferase (ALT), aspartate transaminase (AST), albumin (ALB), total bilirubin (TBIL), prothrombin time (PT), international normalization ratio (INR), creatinine(Cr), and platelet (PLT)), was extracted from their medical records。

All subjects were assigned into the SCT group or Non-SCT group according to whether they received HUCB-MSCT during hospitalization. Whether to accept HUCB-MSCT depends on the patient’s own will, there was no subjective artificial choice of doctors. Informed consent was signed prior to stem cell treatment. The survival outcomes and incidence of HCC were obtained by case records and confirmed by telephone calls. Follow-up was performed from the time of hospitalization to October 30, 2021, or the date of death or liver transplantation. HCC was diagnosed according to the Expert Consensus on Standardization of the Management of HCC in China. Abdominal B ultrasonography and CT or MRI scans were performed to exclude other disorders.

All patients in the study received standard clinical treatments (including albumin supplementation, coagulation correction, liver protection, antiviral treatment, and necessary anti-infective treatment) and management of complications such as ascites, variceal bleeding, infection, and hepatorenal syndromes according to updated guidelines.

### Outcomes

The primary outcome was the overall survival. The secondary outcomes were 3-year and 5-year survival rates and the occurrence rates of HCC. Overall survival was defined as the time from the start of the study to the death of a case.

### Statistical Analysis

Statistical analysis was conducted using the R software (version 4.0.2). Each variable was tested for normality using the Shapiro–Wilk test. Continuous variables were expressed as mean ± standard deviation (SD) if normally distributed or median (interquartile) if not normally distributed and were analyzed using a two-sample *t*-test or Wilcoxon rank sum test, as appropriate. Sex and etiology were expressed as the number of patients (percentage) and tested with the *χ*^2^ test or Fisher’s exact test, as appropriate. A ratio of 1 transplantation patient for every 2 non-SCT patients was achieved by propensity score matching using the MatchIt R package. Thirteen factors were included in the propensity score matching analysis including sex, age, etiology, alanine aminotransferase, aspartate aminotransferase, total bilirubin, albumin, international normalized ratio, serum sodium, creatinine, platelets, CTP score, and MELD score. Univariate and multivariate Cox proportional hazard regression analyses were performed to identify significant demographic and clinical characteristics. Survival curves were plotted using the Kaplan–Meier method and analyzed using the log-rank test. All analyses were performed as two-sided tests with a 0.05 level of significance.

## Results

A total of 248 patients with DLC were initially enrolled, and 47 patients were excluded. Finally, a total of 201 subjects were enrolled, including 36 patients undergoing HUCB-MSCT and 165 patients without transplantation. Except for albumin (ALB) levels, there were no significant differences between the non-SCT group and the SCT group (all *P* > 0.05) before PSM. The bias was addressed by limiting the ratio of cases to non-SCTs to 1 to 2, which resulted in 36 patients in SCT group and 72 patients in non-SCT group with comparable baseline characteristics (Table 1).

**Table 1:** Baseline characteristics of two groups before and after PS matching

	Before PS matching			After PS matching		
	Non-SCT (*n* = 165)	SCT (*n* = 36)	*P* value	Non-SCT (*n* = 72)	SCT (*n* = 36)	*P* value
Sex, *n* (%)			0.405			0.736
Male	117 (70.91)	28 (77.78)		58(80.56)	28 (77.78)	
Female	48 (29.09)	8 (22.22)		14(19.44)	8 (22.22)	
Age (years)	58 (46–65)	51.5 (45–63)	0.091	51.44 ± 11.72	52.11 ± 12.02	0.785
Cause, *n* (%)			0.792			0.728
HBV infection	142 (86.06)	32 (88.89)		66 (91.67)	32 (88.89)	
Others	23 (13.94)	4 (11.11)		6 (8.33)	4 (11.11)	
ALT (U/L)	41 (25–65)	32 (22.25–54.15)	0.246	45.5(28–69.75)	32 (22.25–54.15)	0.081
AST (U/L)	66 (45–110)	50.5 (40.75–80.25)	0.075	60(42.75–102.75)	50.5 (40.75–80.25)	0.227
ALB (g/L)	31(27.3–33.6)	32.95(29.88–34.10)	0.043	32.85(29.77–34.10)	32.85 (29.77–34.10)	1.000
TBIL (umol/L)	40.1(35.2–62.9)	38.35(25.20–58.17)	0.214	37.8(33.28–43.25)	38.35(25.20–58.17)	0.830
PT(s)	15.2(13.5–18.0)	15.60 (14.35–18.42)	0.272	14.9(13.55–17.62)	15.6(14.35–18.42)	0.234
INR	1.23 (1.08–1.42)	1.27 (1.16–1.47)	0.204	1.19(1.10–1.43)	1.27(1.16–1.47)	0.194
Cr (umol/L)	68.5 (58.0–86.7)	74.75 (64.95–84.55)	0.595	67.9(60.08–80.00)	74.75(64.95–84.55)	0.262
PLT (*10^9/L)	73 (54–124)	72.50 (51.75–123.50)	0.577	69.5(46–114)	72.5(51.75–123.50)	0.767
CTP score	9 (7–10)	7 (7–10)	0.239	8(7–10)	7(7–10)	0.9611
CTP class			0.816			0.464
B	102(61.81)	23(63.89)		51(70.83)	23(63.89)	
C	63(38.18)	13(36.11)		21(29.17)	13(36.11)	
MELD score	11.78(10.02–14.85)	11.19(9.44–13.82)	0.449	10.84(10.11–13.50)	11.19(9.44–13.82)	0.953

Data were expressed as number (%), mean ± standard deviation (SD), or median (IQR). *HBV*, hepatitis B virus; *ALT*, alanine aminotransferase; *AST*, aspartate transaminase; *ALB*, albumin; *TBIL*, total bilirubin; *PT*, prothrombin time; *INR*, international standard ratio; *Cr*, creatinine; *PLT*, platelet; *CTP*, Child-Turcotte-Pugh; *MELD*, model for end-stage liver disease

**Fig. 1:**
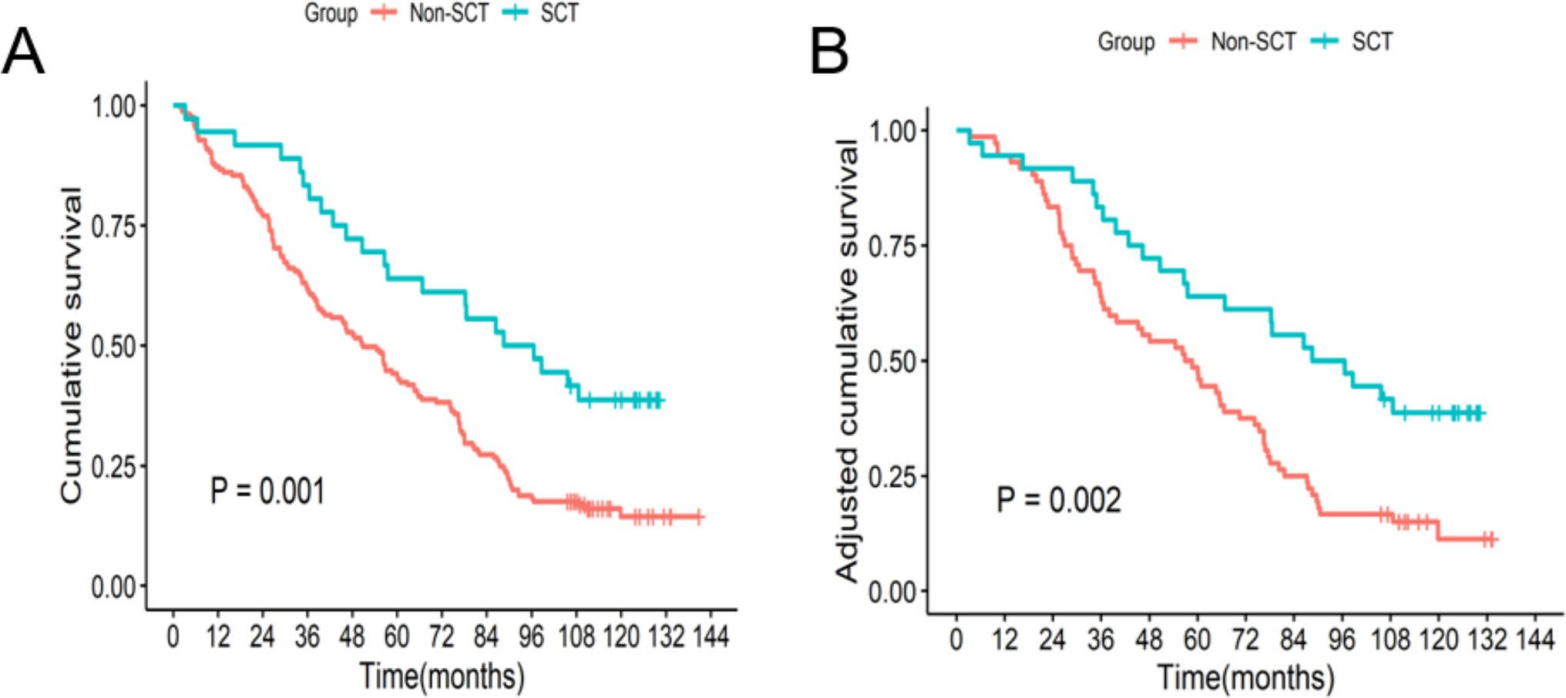
Overall survival between the two groups. **A** Survival curve of the entire cohort; **B** survival curve of the matched cohort

Before PSM, the overall mortality was 80.1% (161/201) in all study subjects, with 61.1% (22/36) mortality in the SCT group and 84.2% (139/165) in the Non-SCT group (*P* = 0.002). The median OS for the SCT group was 92.50 (95% CI, 57.5–NA) months, and 50.80 (95%CI, 39.8–64.5) months for the non-SCT group. After PSM, the overall mortality was 74.1% (80/108) in study subjects, with 61.1% (22/36) mortality in the SCT group and 80.6% (58/72) in the non-SCT group (*P* = 0.030). The median OS for SCT group was 92.50 (95%CI, 57.5–NA) months, and for Non-SCT group was 57.7 (95%CI, 38.0–74.2) months. The 3-year and 5-year survival rates between the two groups were 83.3% vs. 61.8% and 63.9% vs. 43.6%, respectively. After PSM, the 3-year and 5-year survival rates between the two groups were 83.3% vs. 63.9% and 63.9% vs. 47.2%, respectively. The Kaplan–Meier survival curves before and after PSM were significantly higher in the SCT group than in the non-SCT group (all *P* < 0.05) (Fig. 1). By multivariate regression analysis, HUCB-MSCT treatment was found to be an independent benefificial factor for patient OS (Table 2).

**Table 2:** Cox regression analysis of risk factors of survival in the entire cohort

	Univariate analysis			Multivariate analysis		
	Hazard ratio	95% CI	*P* value	Hazard ratio	95% CI	*P* value
Sex	1.29	0.75–2.23	0.353	-	-	-
Age	0.99	0.97–1.01	0.201	-	-	-
Cause	0.95	0.46–1.96	0.884	-	-	-
ALT	1.00	1.00–1.00	0.582	-	-	-
AST	1.00	1.00–1.00	0.692	-	-	-
ALB	1.00	0.94–1.06	0.931	-	-	-
TBIL	1.00	1.00–1.00	0.472	-	-	-
PT	1.04	1.00–1.09	0.057	-	-	-
INR	1.60	0.9–2.83	0.110	-	-	-
Cr	1.00	0.99–1.01	0.488	-	-	-
PLT	1.00	0.99–1.00	0.597	-	-	-
CTP score	1.02	0.91–1.15	0.688	-	-	-
CTP class	1.21	0.77–1.90	0.414	-	-	-
MELD	1.02	0.98–1.06	0.399	-	-	-
HUCB-MSCT	0.47	0.29–0.76	0.002	0.47	0.29–0.76	0.002

*HUCB-MSCT*, human umbilical cord blood -derived mesenchymal stem cell transplantation; *ALT*, alanine aminotransferase; *AST*, aspartate transaminase; *ALB*, albumin; *TBIL*, total bilirubin; *PT*, prothrombin time; *INR*, international standard ratio; *Cr*, creatinine; *PLT*, platelet; *CTP*, Child-Turcotte-Pugh; *MELD*, model for end-stage liver disease

**Fig. 2:**
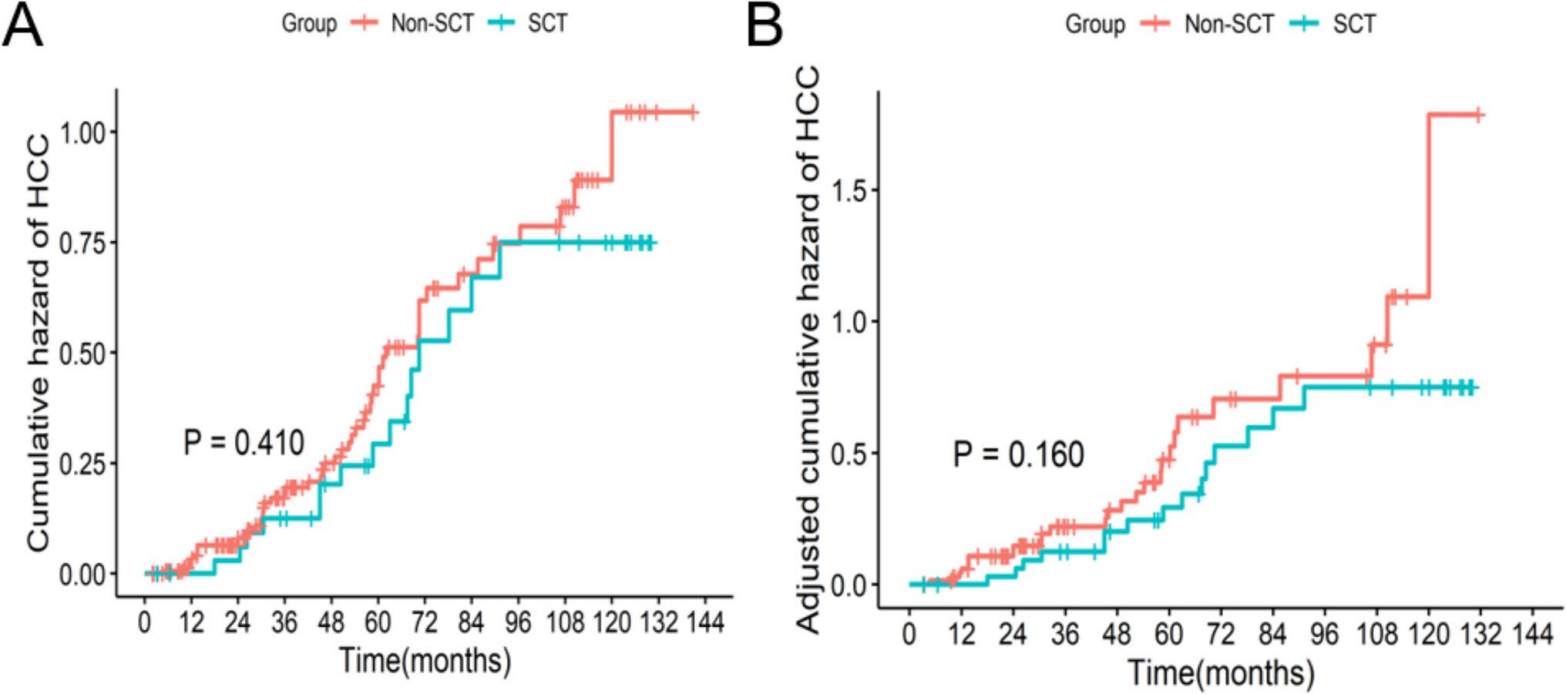
Incidence of HCC between the two groups. **A** Cumulative risk function of HCC of the entire cohort; **B** cumulative risk function of HCC of the matched cohort

In the entire cohort, the overall incidence of HCC was 33.3% (67/201), with 41.7% (15/36) incidence in the SCT group and 31.5% (52/165) in the non-SCT group (*P* = 0.242). In the matched cohort, the overall incidence of HCC was 32.4% (35/108), with 41.7% (15/36) incidence in the SCT group and 27.8% (20/72) in the non-SCT group (*P* = 0.146). No significant difference was found in the cumulative risk function of HCC between the two groups before and after PSM (all *P* > 0.05) (Fig. 2).

## Discussion

This study demonstrates that HUCB-MSCT can improve survival outcomes in patients with liver cirrhosis. Additionally, the results found that HUCB-MSCT did not increase the risk of HCC in these patients. We therefore conclude that HUCB-MSCT may be a safe and effective method for the treatment of liver cirrhosis, both in the long- and short-term.

A large-scale meta-analysis of randomized non-SCT led trials (RCTs) evaluating the therapeutic effects and safety of stem cell therapy for chronic liver disease (CLD) revealed that stem cell therapy is a safe and effective therapeutic option for CLD.^18^ HUCB-MSCTs have shown great potential in regenerative medicine due to their multilineage differentiation potential, low immunogenicity, and self-renewal ability.^19,20^ Many recent studies have shown that patients with decompensated cirrhosis receive significant short-term benefits from HUCB-MSCT.^18–20^ However, there was a lack of data demonstrating the long-term benefit of HUCB-MSCT in patients with decompensated cirrhosis. In addition, most previous clinical studies only evaluated the safety of this procedure in the short-term. In view of the possible tumorigenicity of stem cell therapy, long-term follow-up observation was necessary.

In contrast with this report, a recent meta-analysis which examined clinical outcomes of stem cell transplantation from various human tissue sources in cirrhotic patients showed no significant difference in the mortality between the treatment and non-SCT groups, and concluded that HUCB-MSCT could improve liver function but appeared to not be significant in increasing the survival in cirrhotic patients.^21^ However, in another clinical study, autologous transplants of peripheral blood stem cells significantly improved long-term survival as compared to the non-SCT group.^22^ This difference in outcome may be related to differing source of cells, methods of transplantation, and degrees of liver cirrhosis.

Patients with cirrhosis are at increased risk of developing HCC. It was an open question if stem cell transplantation could potentially further increase the incidence of HCC in patients with cirrhosis. A recent study recommended close monitoring of patients undergoing autologous SCT for HCC.^23^ However, this conflicts with another report and our study which did not find a difference in HCC prevalence between standard medical treatment and peripheral blood stem cells treatment.^22^ In a high carcinogenic risk liver cirrhosis mouse model, no significant differences were observed between the non-SCT and bone marrow stem cell treatment groups in the incidence, number, or mean size of lesions and tumors on histological evaluation.^24^ Our long-term follow-up results showed that HUCB-MSCT did not increase the risk of HCC. Moreover, research has shown that stem cell may improve the immunologic status and microenvironment of target tissues and therefore potentially may delay HCC development.^25^ However, further studies are necessary to fully understand the effects of stem cell treatment on HCC development.

However, there are several limitations of this study. Firstly, it was a retrospective study and selection biases may have existed since this study was subject to the inherent limitations associated with retrospective analyses. Secondly, as a single-center study, the sample size of this study was not large enough to draw firm conclusions. In the future, large-scale and prospective studies are required to clarify the long-term survival benefit and safety of HUCB-MSCTs in patients with decompensated cirrhosis.

In conclusion, this study suggests HUCB-MSCT may improve long-term survival without increasing the risks of HCC in patients with decompensated cirrhosis.

## Data Availability

All data generated or analysed during this study are included in this published article.

## Abbreviations

HBV: Hepatitis B virus
ALT: Alanine aminotransferase
UCMSCs: Umbilical cord mesenchymal stem cells
ACLF: Acute-on-chronic liver failure
TBIL: Total bilirubin
PTA: Prothrombin activity
MELD: Model for end-stage liver disease
